# Changes in the cervicovaginal microbiota composition of HPV16‐infected patients after clinical treatment

**DOI:** 10.1002/cam4.4801

**Published:** 2022-05-15

**Authors:** Chao Li, Zhenbo Zhang, Yixia Yang, Hong Liao

**Affiliations:** ^1^ Shanghai Key Laboratory of Maternal Fetal Medicine, Shanghai First Maternity and Infant Hospital Tongji University School of Medicine Shanghai China; ^2^ Reproductive Medicine Center, Department of Obstetrics and Gynecology, Shanghai General Hospital Shanghai Jiaotong University Shanghai China; ^3^ Department of Lab Medicine, Shanghai First Maternity and Infant Hospital Tongji University School of Medicine Shanghai China

**Keywords:** cervical diseases, cervicovaginal microbiota, community state type, HPV16, *Lactobacillus*, pyrosequencing

## Abstract

**Background:**

High‐risk human papillomavirus (hrHPV) infection is a key factor that alters cervicovaginal microbiota patterns and causes cervical intraepithelial neoplasias (CINs) or even cervical cancer. Although local excisional treatment can clear hrHPV infection and restore the cervicovaginal microbiota, it is unclear which cervicovaginal microbiota represents recovery. Our objective was to describe the cervicovaginal microbiota before and after treatments and to assess the association between the microbiota and HPV persistence.

**Results:**

A cohort of 91 participants was classified into four groups (healthy control women and HPV16‐infected women with CIN I, CIN II/III, and squamous cell carcinoma [SCC]). Endocervical swabs were collected 3 months prior to treatment and at 3 months post‐treatment for bacterial 16S rRNA gene pyrosequencing and for HPV DNA testing. There was an increase in the number of *Lactobacillus* bacterial species present after the clinical treatments, and the community state type (CST) profiles were shifted from dysbiotic CSTs II and IV to *Lactobacillus*‐dominated CSTs I and III. Specifically, the composition of *Geobacter* and *Prevotella* before treatment and *Lactobacillus secaliphilus* after treatment might have been related to CIN I, the composition of *Burkholderia* before treatment and *Lactobacillus iners* after treatment might have been related to CIN II/III, and the composition of *Atopobium* and *Aerococcus* before treatment and Bacilli after treatment might have been related to SCC. Further functional predictions revealed that the composition differences were linked to infectious disease‐ and cancer‐related genes.

**Conclusion:**

Our study provides an illustration of the changes in CSTs and the cervicovaginal microbiota before and after HPV16 clearance in each disease state.

## Graphical abstract

This study interpreted the differential profiles of cervicovaginal microbiota and CSTs between HC women and HPV16‐infected women with CIN I, CIN II/III or SCC. The relationship between HPV16 clearance and cervicovaginal microbiota composition in each disease stage before and after clinical treatments was also established.

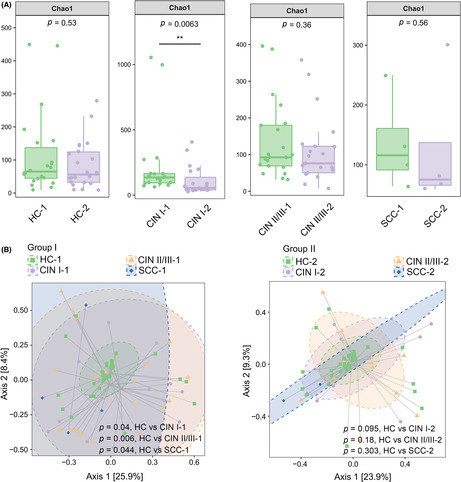

## 1 INTRODUCTION

Cervical cancer is the third most common cancer diagnosed among women worldwide and is almost exclusively attributed to a group of high‐risk (hr) human papillomaviruses (HPVs), such as HPV16 and 18.^1^, ^2^ HPV16 is responsible for approximately 50% of cervical cancers.^3^ In general, the majority of infections are cleared spontaneously by the immune system. A fraction of patients with persistent hrHPV develop cervical intraepithelial neoplasias (CINs) or even progress to cervical cancer.^4^ Thus, there could be influences other than the HPV subtype on the progression of CIN to cervical cancer.

Emerging evidence supports the idea that the cervicovaginal microbiota may play a vital role in influencing HPV acquisition, persistence, and progression to cervical dysplasia and malignancy.^5^ On the one hand, changes in cervicovaginal microbiota composition are likely correlated with the acquisition of hrHPV types. Many cross‐sectional and longitudinal studies have noted that individuals with non‐*Lactobacillus* species or *Lactobacillus iners* compositions (such as found in community state type [CST] IV) had over two times higher chances of being infected with hrHPVs.^6^ Similarly, in our previous study, a composition of *Oribacterium*, *Thermus* and *Lachnobacterium*, *Motilibacter* and *Paludibaculum,* and *Litorilinea* with a lack of *L. iners* might have been related to the infection of HPV16, 52, and 58, respectively.^2^ On the other hand, the degree of microbiota diversity affects the pathogenicity of HPV infection. The vaginal microbiota is the first line of defense against HPV infection, and it is capable of producing lactic acid and H_2_O_2_ to protect the cervical mucosa against viral invasion.^7^, ^8^ A negative association between persistent HPV infection and CIN with *Lactobacillus* dominance, with the exception of *L. iners*, has been highlighted.^9^ In addition, increased cervicovaginal microbiota diversity is positively associated with CIN disease progression. Mitra et al. and others have reported that the paucity of *Lactobacillus* with concomitant occupation by *Gardnerella vaginalis*, *Atopobium vaginae*, and *L. iners* is related to CIN risk.^10^, ^11^, ^12^, ^13^, ^14^, ^15^

Although previous findings have been consistent and highly suggestive of an altered vaginal microbiota, few studies have explored the impact that the cervicovaginal microbiota composition may have on the clinical outcome of CIN/cervical cancer after HPV clearance. Two recent studies examined samples from women before and after the loop electrosurgical excision procedure (LEEP) and revealed that the reduction in *Prevotella*, *Leptotrichia*, and *Clostridium* abundances^16^ and the concomitant increase in a *Lactobacillus*‐dominated microbiota could promote clearance after HPV infection.^17^ This observation is similar to that of a recent report on clinical outcomes in women with untreated CIN II; women carrying a *Lactobacillus*‐dominant microbiota at the beginning were more prone to have regressive disease, whereas *Lactobacillus* depletion with increased abundance of anaerobes was associated with CIN II persistence and slower regression.^4^ Nevertheless, it should be noted that a systematic assessment of specific bacterial patterns that influence the microbial recovery of CIN I, CIN II/III, and cervical cancer patients after clinical treatment is lacking.

In this study, we evaluated the microbiota composition of 25 healthy control (HC) women with normal cytology (chronic cervicitis) and 66 HPV16‐infected women with CIN I, CIN II/III, and squamous cell carcinoma (SCC) pathology. The distribution of bacteria was identified and classified using a barcoded 16S rRNA (V3–V4) pyrosequencing approach. We first examined the temporal relationships between the cervicovaginal microbiota and the natural history of the HC women and between the cervicovaginal microbiota and CIN I, CIN II/III, or SCC before and after 3 months of treatment. Then, we identified specific microbiota patterns at each disease stage and characterized the microbial species. Finally, we determined the relationship between the differentially abundant microbial species and microbial recovery after HPV16 clearance.

## 2 MATERIALS AND METHODS

### 2.1 Sample collection and study design

Four hundred thirty‐seven subjects were enrolled at Shanghai First Maternity and Infant Hospital between October 2017 and June 2019. The inclusion criteria of the participants were as follows: (a) no pregnancy, lactation, or menstruation at the time of sampling; (b) no vaginal intercourse or vaginal lavage within the last 3 days; (c) no use of antibiotics/probiotics or barrier contraceptive products in the past month; (d) no HIV or hepatitis B/C positivity; and (e) no previous history of endocrine or autoimmune disorders or malignant tumors.

Two swabs were used to collect cervicovaginal samples from each woman. One swab was collected 3 months before clinical treatment (regular follow‐up for HCs; antiviral treatment [recombinant human interferon α2a vaginal suppository] for CIN I, cervical excision by LEEP for CIN II/III, and radical hysterectomy for SCC patients), and the second was collected 3 months after treatment (when the women were found to be negative for HPV16). Cervical cells were collected as described previously.^2^ For the SCC patients treated with radical hysterectomy, the sample was obtained from the upper vagina. One half of the swab was used for the detection and typing of HPV DNA, and the rest was stored at −80°C for subsequent pyrosequencing.

### 2.2 HPV genotyping

HPV genotyping was performed as previously described^2^ using the HPV GenoArray test kit. The HPV blot contained 21 types of genotypes, including 14 high‐risk types, two intermediate‐risk types, and five low‐risk types, all of which are common in the Chinese population. In each reaction, the absence of HPV DNA contamination was confirmed by assessment of HPV L1, and an internal control of human α‐globin was also used.

### 2.3 DNA extraction and sequencing of the 16S rDNA amplicon

Extraction of bacterial DNA was performed as described.^2^ The V3 and V4 hypervariable regions of the bacterial 16S rRNA gene were amplified using the primer pairs 338F (5′‐ACTCCTACGGGAGGCAGCA‐3′) and 806R (5′‐GGACTACHVGGGTWTCTAAT‐3′) fused to a 6‐bp barcode (Table S1). The purified amplicons were mixed in equal amounts, and sequencing was performed at Shanghai Personal Biotechnology Co., Ltd. (Shanghai, China) using the Illumina MiSeq platform (MiSeq Reagent Kit v3).

### 2.4 Sequence analysis

The resulting microbial sequence data were analyzed using the Quantitative Insights into Microbial Ecology 2 (QIIME2) pipeline and R packages (v3.2.0).^18^ The final obtained high‐quality sequences were clustered into operational taxonomic units (OTUs) at 97% identity by UCLUST.^19^ The NCBI NT_database (https://www.ncbi.nlm.nih.gov/nuccore?term) was used for sequence alignment of the nonsingleton OTUs. α diversity metrics (Shannon, observed species, Chao1, Simpson, and Good's coverage) and β diversity metrics (weighted UniFrac, unweighted UniFrac, Bray–Curtis dissimilarity, and Jaccard distance) were investigated using the diversity plugin with samples rarefied to 8498 sequences per sample.^20^ Taxonomy was assigned to OTUs using MEGAN4 and GraPhlAn.^21^, ^22^

### 2.5 Statistical analyses

OTU‐level α diversity indices were calculated using the OTU table described above in QIIME2 and visualized as box plots. OTU‐level ranked abundance curves were plotted to explain the richness and evenness of OTUs. β diversity analysis was carried out to estimate the structural similarity of bacterial communities among groups and with principal coordinate analysis (PCoA).^23^ A Venn diagram was created to show the shared and unique OTUs or genera among groups regardless of their relative abundances.^24^ Linear discriminant analysis effect size (LEfSe) was used to detect differentially abundant taxa among groups for biomarker discovery using the default parameters.^25^ Orthogonal partial least squares discriminant analysis (OPLS‐DA) was employed to discriminate the microbiota variation across groups.^26^ The functional abundance of the microbiota was computed using Phylogenetic Investigation of Communities by Reconstruction of Unobserved States 2 (PICRUSt2) in the Kyoto Encyclopedia of Genes and Genomes (KEGG) database (https://www.kegg.jp/).

## 3 RESULTS

### 3.1 Participant population

After HPV genotyping and clinical pathological diagnosis of the recruited women, 91 women, 66 of whom were singly infected with HPV16, were chosen as the cohort and classified into four groups: HC women (*n* = 25), HPV16‐infected women with CIN I (*n* = 26), HPV16‐infected women with CIN II/III (*n* = 34), and HPV16‐infected women with SCC (*n* = 6) (Table S1). The general characteristics of the study participants are shown in Table 1. There were no significant differences between women with and without cervical lesions except for a difference in the age of first sexual experience in SCC patients (*p =* 0.041).

**TABLE 1:** General characteristics of the study subjects (*n* = 91)

	HC (*n* = 25)	CIN I (*n* = 26)	*p* value vs HC	CIN II/III (*n* = 34)	*p* value vs HC	SCC (*n* = 6)	*p* value vs HC
Age (years)							
≤29	3 (12.0)	4 (15.4)	0.21	6 (17.6)	0.166	0 (0.0)	0.067
30–39	15 (60.0)	12 (46.2)		14 (41.2)		1 (16.7)	
40–49	3 (12.0)	7 (26.9)		9 (26.5)		2 (33.3)	
50–59	4 (16.0)	1 (3.8)		2 (5.9)		2 (33.3)	
≥60	0 (0.0)	2 (7.7)		3 (8.8)		1 (16.7)	
Sexual partners							
1–5	23 (92.0)	22 (84.6)	0.668	31 (91.2)	0.999	5 (83.3)	0.488
≥6	2 (8.0)	4 (15.4)		3 (8.8)		1 (16.7)	
Age at sexual debut							
<20	2 (8.0)	1 (3.8)	0.471	3 (8.8)	0.971	3 (50.0)	**0.041**
20–29	22 (88.0)	25 (96.2)		30 (88.2)		3 (50.0)	
≥30	1 (4.0)	0 (0.0)		1 (2.9)		0 (0.0)	
No. of childbirths							
0	5 (20.0)	3 (11.5)	0.191	2 (5.9)	0.068	0 (0.0)	0.092
1	11 (44.0)	18 (69.2)		25 (73.5)		4 (66.7)	
2	9 (36.0)	5 (19.2)		6 (17.6)		1 (16.7)	
≥3	0 (0.0)	0 (0.0)		1 (2.9)		1 (16.7)	
No. of abortions							
0	19 (76.0)	15 (57.7)	0.343	20 (58.8)	0.147	4 (66.7)	0.525
1	4 (16.0)	6 (23.1)		13 (38.2)		2 (33.3)	
≥2	2 (8.0)	5 (19.2)		1 (2.9)		0 (0.0)	

Abbreviations: CIN, cervical intraepithelial neoplasia; HC, healthy control; SCC, squamous cell carcinoma.

*p* values were determined by the chi‐squared test or *t‐*test.

*p* < 0.05 was considered to indicate statistical significance in bold.

After barcode‐based pyrosequencing of cervicovaginal secretion samples from the cohort of 91 individuals, a total of 6,733,474 high‐quality sequences, with an average of 36,795 reads, for each sample were identified (Figure S1 and Table S2). The average length of sequence reads was 425 bp, and they were clustered into 11,902 OTUs using UCLUST.^27^ Further removal of singleton OTUs resulted in 3217 taxa per group on average (Table S3).

### 3.2 Overall bacterial structure differences

To characterize the impact of clinical treatments on cervicovaginal microbiota recovery, we used *Lactobacillus* as the standard since high proportions of *Lactobacillus* are frequently considered to represent a “normal” bacterial community for most healthy women.^28^ As shown in Figure S2A, an increased abundance of *Lactobacillus* was observed in approximately 56% (14/25) of the HC women with regular follow‐up. With respect to those patients who underwent clinical treatments, the proportions were 73.1% (19/26), 61.8% (21/34), and 66.7% (4/6) for CIN I, CIN II/III, and SCC patients, respectively (Figure S2B–D).

**FIGURE 1:**
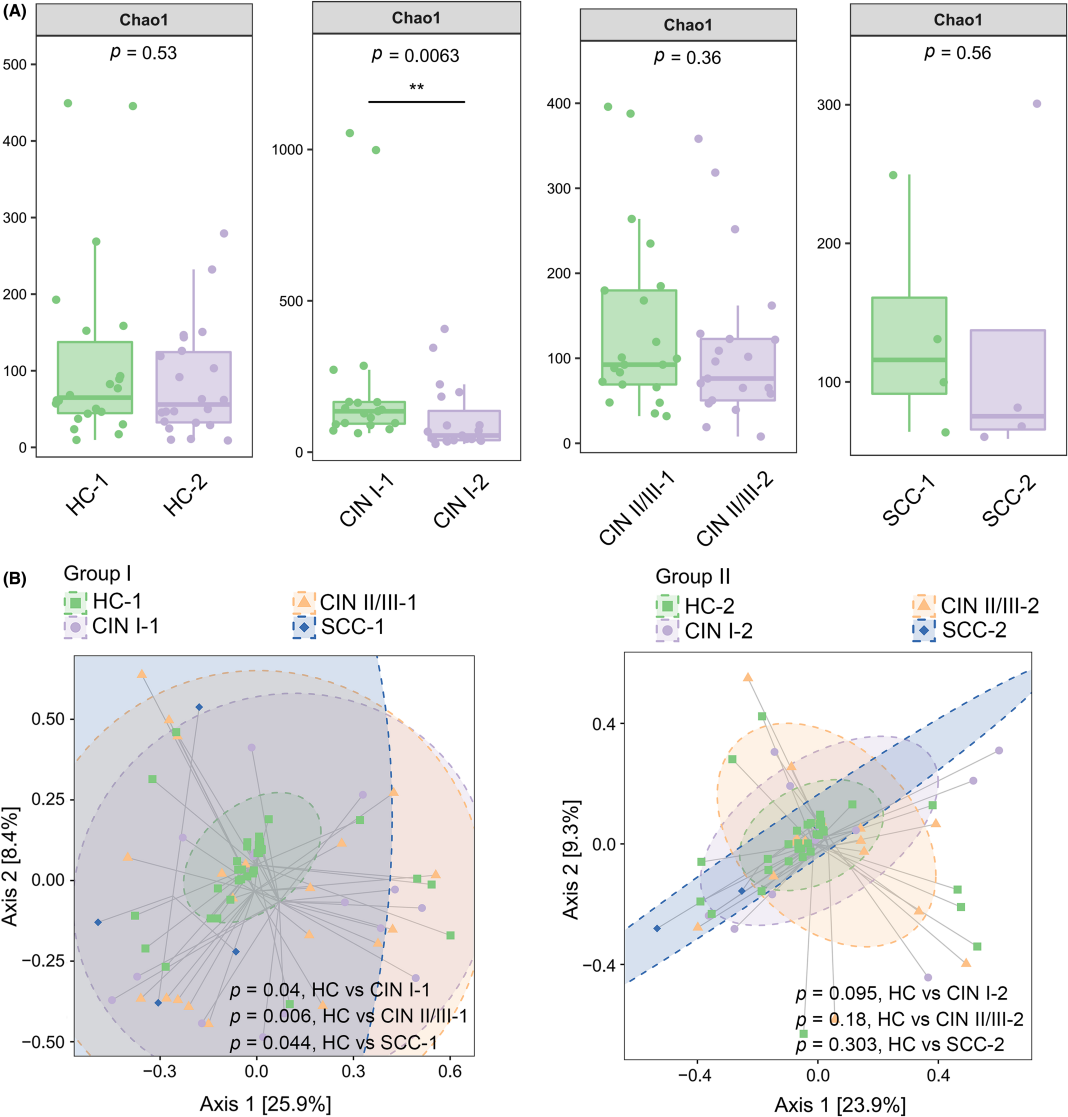
Analysis of α diversity and β diversity in 66 patients before and after clinical treatment. (A) Analysis of the Chao1 α diversity at the genus level. HC‐1, CIN I‐1, CIN II/III‐1, and SCC‐1 indicate that the samples were collected before clinical treatment; HC‐2, CIN I‐2, CIN II/III‐2, and SCC‐2 indicate that the samples were collected after clinical treatment. (B) A total of 132 samples were clustered into Group I (before treatment) and Group II (after treatment), which were then analyzed by weighted UniFrac distance‐based PCoA at the genus level. Shaded ellipses represent 95% confidence intervals. Mann–Whitney *U* test *p* values are shown

To investigate the specific bacteria that participate in microbial recovery after HPV16 clearance, a total of 66 patients (132 samples; Figure S3 and Table S4) with an increased abundance of *Lactobacillus* were selected for further analysis. The Chao1 α diversity index was much lower in the CIN I‐2 group (*p =* 0.0063, CIN I‐1 vs CIN I‐2; “‐1” indicates before treatment and “‐2” indicates after treatment; Figure 1A). The observations are in line with previous reports,^16^, ^17^ suggesting the possible deficiency of healthy microbiota constituents, such as *Lactobacillus*, in these patients. Although the CIN II/III‐2 and SCC‐2 groups exhibited a downward trend, the differences did not reach statistical significance, indicating poorer recovery of the cervicovaginal microbiota in patients with CIN II/III and SCC.

To evaluate the difference in bacterial communities at different stages of cervical diseases, PCoA based on the weighted UniFrac distances was carried out to separate the 132 samples into two groups (Figure 1B). As shown in Group I, the microbiota of the HC women was dramatically different from those in CIN I‐1, CIN II/III‐1, or SCC‐1 women (*P =* 0.04, HC vs. CIN I‐1; *p =* 0.006, HC vs CIN II/III‐1; *p =* 0.044, HC vs SCC‐1), while the latter three groups were somewhat similar (*p =* 0.32, CIN I‐1 vs CIN II/III‐1; *p =* 0.321, CIN I‐1 vs SCC‐1; *p =* 0.987, CIN II/III‐1 vs SCC‐1) and partly overlapped with each other. In contrast, no obvious difference in the community structures was found between the HC and CIN I‐2, CIN II/III‐2, or SCC‐2 women (*p =* 0.095, HC vs CIN I‐2; *p =* 0.18, HC vs CIN II/III‐2; *p =* 0.303, HC vs SCC‐2) in Group II. These findings confirm that Group I women had a cervicovaginal microbiota community structure associated with different stages of cervical diseases, while the microbiota community structure is more similar among Group II women.

### 3.3 Distribution and abundance of the bacteria

In the four groups, five phyla, namely, Firmicutes, Bacteroidetes Proteobacteria, Actinobacteria, and Fusobacteria, were dominant, together accounting for 99.01%, 98.94%, 98.28%, and 98.39% of the total reads in the HC, CIN I, CIN II/III, and SCC groups, respectively (Table S5). Firmicutes and Proteobacteria were the two most dominant phyla in samples from the HC (39/44), CIN I (28/38), CIN II/III (32/42), and SCC (8/8) groups. Higher abundances of Proteobacteria and Bacteroidetes were observed in women before clinical treatment, whereas the abundance of Firmicutes displayed the opposite trend (Figure S4; Table S6). In addition, Proteobacteria was more abundant in patients with SCC either before or after treatment than in those with CIN I and CIN II/III; the opposite result was observed regarding Bacteroidetes.

### 3.4 Coabundant bacteria enriched in each group

Venn diagrams at the genus level were generated to identify common coabundant bacteria. Of the 435 genera identified in Group I, the number of genera in the HC, CIN I‐1, CIN II/III‐1, and SCC‐1 groups was 307, 348, 270, and 143, respectively (Figure S5A). In particular, 126 genera were shared among all four subgroups, with the top three being *Lactobacillus* (49.7% of total abundances), *Burkholderia* (13.9%), and *Pseudomonas* (11.1%), comprising 74.7% of all the genera (Figure S6A; Table S7). Similarly, 395 genera were identified from Group II, with 306, 255, 213, and 141 genera belonging to HC, CIN I‐1, CIN II/III‐1, and SCC‐1 groups, respectively (Figure S5B). For Group II, 101 genera were shared, and the top three abundant genera were *Lactobacillus* (76.2%), *Pseudomonas* (7.1%), and *Burkholderia* (6.0%), comprising 89.3% of the total genera (Figure S6B; Table S7). There was a reduction in the number of genera (shared genera, genera of each subgroup) after clinical treatment. A tendency toward an increase in the abundance of *Lactobacillus* and a concomitant decrease in the abundance of non‐*Lactobacillus* genera such as *Burkholderia* and *Pseudomonas* were also observed (Figure S6).

### 3.5 Bacterial community types

**FIGURE 2:**
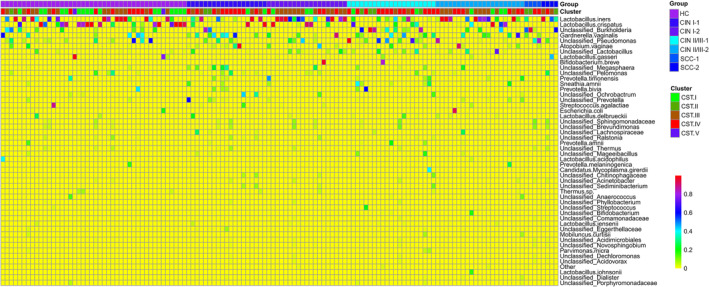
Heatmap analyses of the cervicovaginal microbiota of the 132 samples at the species level. The relative abundances are represented by different colors. CIN I‐1, CIN II/III‐1, and SCC‐1 indicate that the samples were collected before clinical treatment; CIN I‐2, CIN II/III‐2, and SCC‐2 indicate that the samples were collected after clinical treatment

**FIGURE 3:**
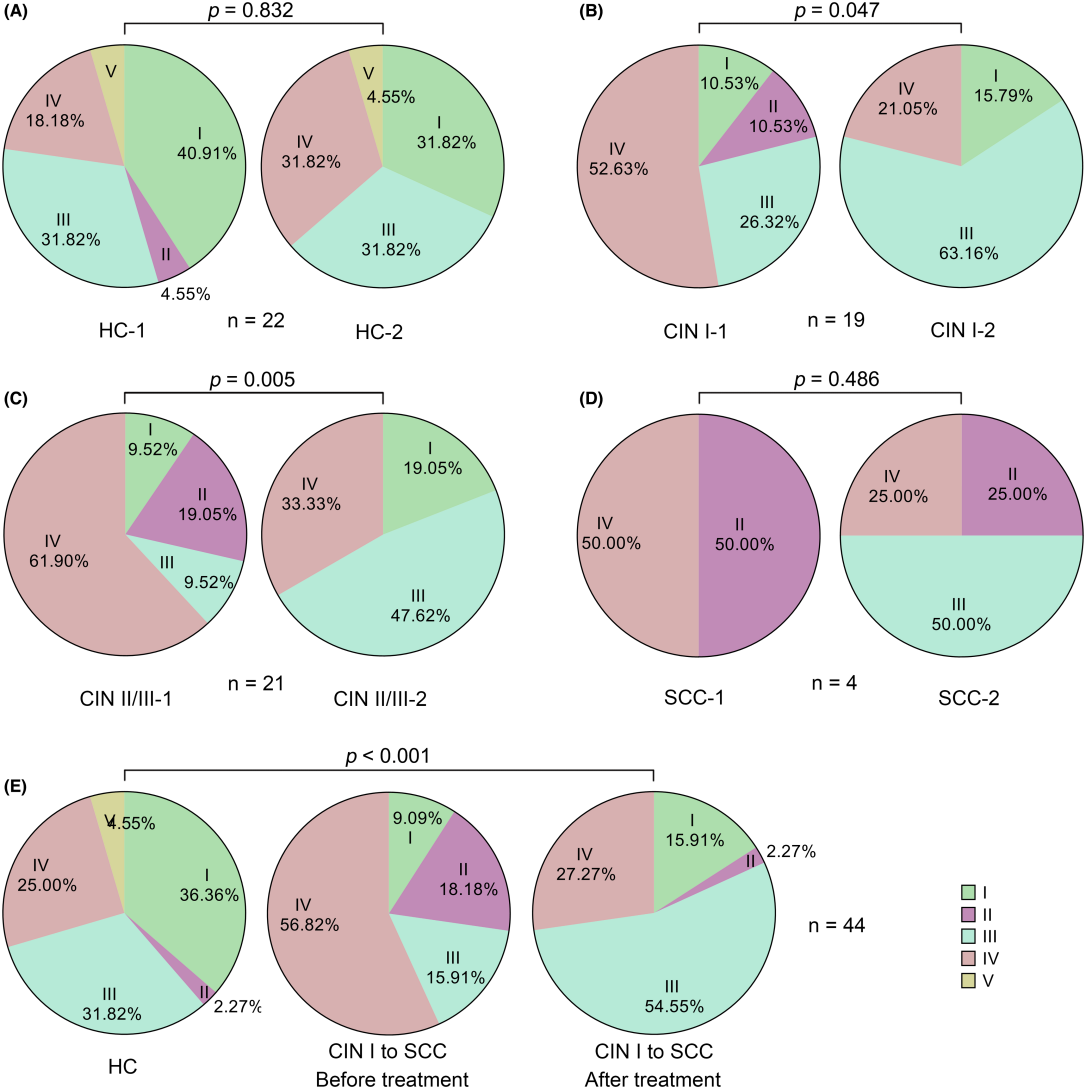
Representation of cervicovaginal bacterial CSTs in the indicated groups. (A) HC. (B) CIN I. (C) CIN II/III. (D) SCC. (E) The change in CSTs before and after treatment. *p* values were determined using the chi‐squared test or Fisher's exact test when the chi‐squared test was not adequate. *p* < 0.05 was considered to indicate statistical significance

The microbiota was classified into five CSTs as previously described^2^ (Figure 2 and Table S8). In brief, *Lactobacillus crispatus* was predominant in CST I; *Burkholderia* was the dominant member in CST II; *L. iners* was predominant in CST III; *G*. *vaginalis*, *Pseudomonas*, *Pelomonas*, *Escherichia coli*, *Atopobium*, *Sneathia amnii*, and *Prevotella* were most abundant in CST IV; and *Lactobacillus gasseri* was predominant in CST V. The resulting CSTs were similar to those in our previous study,^2^ in which none of the CSTs were dominated by *Lactobacillus jensenii*. In the HC group, the majority of the clusters were CST I (40.91%) and III (31.82%) for HC‐1, and CST I (31.82%), III (31.82%), and IV (31.82%) accounted for the majority for HC‐2 (Figure 3A). In the CIN I group, the most abundant cluster was CST IV (52.63%) for CIN I‐1, and CST III (63.16%) was the most abundant cluster for CIN I‐2 (Figure 3B). In the CIN II/III group, the most abundant cluster was CST IV (61.90%) for CIN II/III‐1, and CST III (47.62%) was the most abundant cluster for CIN II/III‐2 (Figure 3C). In the SCC group, the most abundant clusters were CST II and IV for SCC‐1, and CST III was the most abundant cluster for SCC‐2 (Figure 3D).

The prevalence of CSTs also differed among the four groups (Table S9). In the HC group that maintained regular follow‐up, the proportion of the microbiota associated with a healthy status (CST I, CST III, CST V) slightly fluctuated in the HC‐2 samples (68.2%; 15/22), compared to 77.3% (17/22) in the HC‐1 samples. CST I and III (dominated by *Lactobacillus*) existed in 7/19, 4/21, and 0/4 of the CIN I‐1, CIN II/III‐1, and SCC‐1 samples, respectively, while the values were 15/19, 14/21, and 2/4 for the CIN I‐2, CIN II/III‐2, and SCC‐2 samples, respectively. The bacterial communities dominated by CST II and CST IV (non‐*Lactobacillus*) were 12/19, 17/21, and 4/4 and 4/19, 7/21, and 2/4 before and after treatment, respectively. These results suggest that clinical treatments appear to have altered the CSTs toward the healthy types (from CST II and CST IV to CST I and CST III) in the CIN I, CIN II/III, and SCC groups (Figure 3E).

### 3.6 Signature bacteria in each group

**FIGURE 4:**
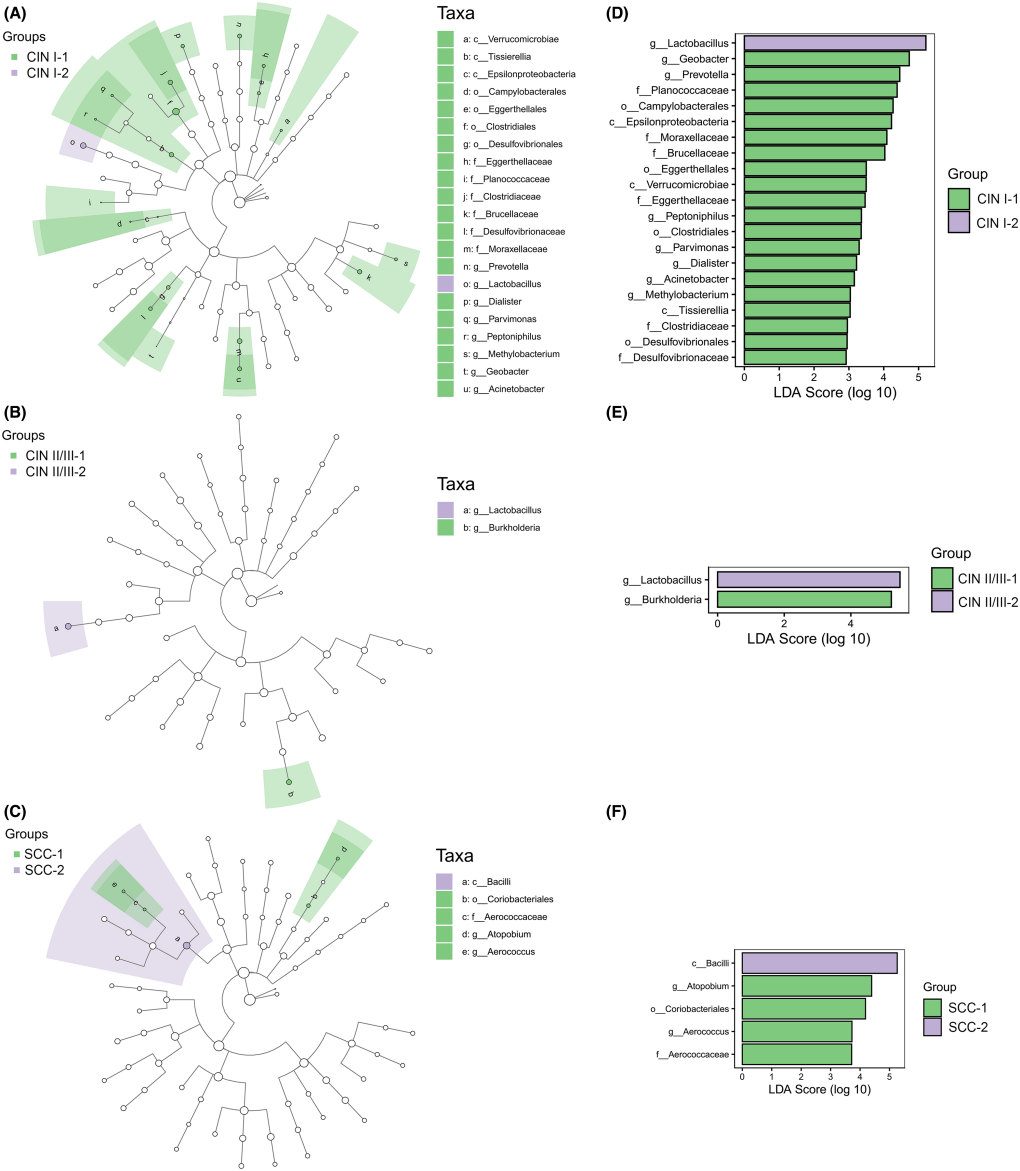
Cervicovaginal microbiota biomarkers of the indicated groups determined by LEfSe analysis. (A–C) The cladogram represents taxa with differential abundances (*p <* 0.05) in the four groups. (D–F) LDA scores as computed indicate differential abundance among groups. Those with an LDA value ≥2 are presented

LEfSe modeling revealed significant differences in the bacterial community compositions among the CIN I, CIN II/III, and SCC groups (Figure 4A–C). In the CIN I group, there were up to 21 different taxa (*p* < 0.05), with the most enrichment in the *Lactobacillus* (CIN I‐2; *p =* 0.026), *Geobacter* (CIN I‐1; *p =* 0.037), and *Prevotella* (CIN I‐1; *p =* 0.048) genera (Table S10). In the CIN II/III group, two different genera, *Lactobacillus* (CIN II/III‐2; *p =* 0.005) and *Burkholderia* (CIN II/III‐1; *p =* 0.002), were significantly overrepresented. In the SCC group, *Atopobium* (SCC‐1; *p =* 0.047) and *Aerococcus* (SCC‐1; *p =* 0.047) were significantly enriched genera (Figure 4D–F). These genera could be regarded as potential biomarkers for predicting the outcome of HPV16 infection‐associated cervical diseases.

Subsequently, the OPLS‐DA model identified 69 genera (variable importance of projection [VIP] score >1; Table S11). Among them, 36, 24, and seven genera were enriched in the CIN I, CIN II/III, and SCC groups, respectively (*p* < 0.05). There were 24 genera selected with a VIP >2, of which five, *Gardnerella*, *Pseudomonas*, *Burkholderia*, *Pelomonas*, and *Lactobacillus*, had a VIP >3. All these genera might serve as disease‐associated bacteria at different stages.

### 3.7 Functional alteration in bacteria in each group

**FIGURE 5:**
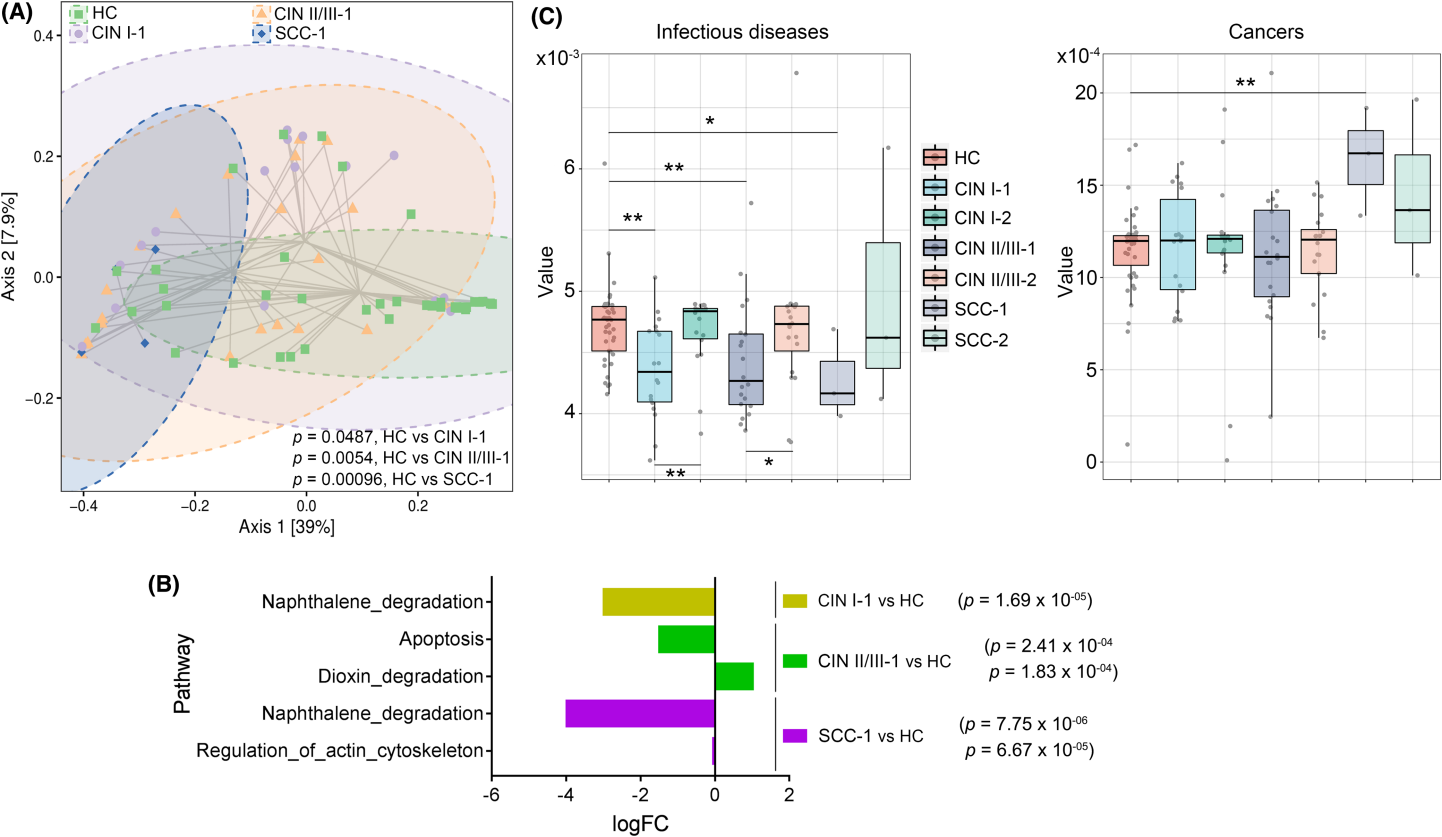
Microbial gene function analysis. (A) PCoA based on the relative abundance of KO groups in Group I. Shaded ellipses represent 95% confidence intervals. (B) Pathway difference analysis within Group I. (C) Predicted functions of the cervicovaginal microbiota connected with human diseases. Left, infectious diseases. Right, cancers. ^**^ *p <* 0.01

A total of 41 classified KEGG Orthology (KO) groups were represented in the dataset (Table S12). PCoA showed significant differences in microbial functions between the HC and other groups (*p =* 0.0487, HC vs CIN I‐1; *p =* 0.0054, HC vs CIN II/III‐1; *p =* 0.00096, HC vs SCC‐1; Figure 5A). Pathway analysis showed that the module of naphthalene degradation was differentially depleted in the CIN I‐1 and SCC‐1 subgroups (Figure 5B). Two other KEGG modules that were depleted in the CIN II/III‐1 and SCC‐1 subgroups were related to apoptosis and regulation of the actin cytoskeleton. These metabolic functions are necessary to maintain a healthy status.^29^, ^30^, ^31^ We observed one module, dioxin degradation that was enriched in CIN II/III‐1, and a lack of this capacity is linked to suppression of the immune system and the formation of cancers.^32^ These results suggested that impairment of the cervicovaginal microbiota might lead to a disease‐related state by interfering with physiological metabolic functions.

Further PICRUSt2 prediction revealed that the subset of cancer‐related genes was more abundant in the SCC‐1 group than in all the other groups, with apparent differences between the SCC‐1 and HC groups (*p =* 0.0084), the SCC‐1 and CIN I‐1 groups (*p =* 0.0072), and the SCC‐1 and CIN II/III‐1 groups (*p =* 0.0051) (Figure 5C). For the subset of infectious disease‐related genes, the distribution densities were differentiated from each other. Significant differences were observed between the HC and CIN I‐1 groups (*p =* 0.001), the HC and CIN II/III‐1 groups (*p =* 0.0085), and the HC and SCC‐1 groups (*p =* 0.048). Functional predictions also revealed significant differences within different groups (*p =* 0.007, CIN I‐1 vs CIN I‐2; *p =* 0.049, CIN II/III‐1 vs CIN II/III‐2). The results suggest that fluctuations in cervicovaginal microbiota composition can induce functional changes in some genes and thus contribute to CINs or even SCC.

## 4 DISCUSSION

Our study includes a systematic evaluation of cervicovaginal microbiota composition in HPV16‐infected women at different disease stages before and after treatment. Although fewer SCC patients (*n* = 6) were recruited, probably because of the poor prognosis after surgery or other reasons such as hospital transfer, approximately equal numbers of HC and CIN participants (25 for HC, 26 for CIN I, and 34 for CIN II/III individuals) were recruited, which provided an appropriate model to assess the relationship between the bacterial composition and the recovery of HPV16‐infected patients after clinical treatment. The number of high‐quality sequences and the sequencing depth were sufficient to detect most of the bacteria.^33^ We identified unique CSTs and bacterial profiles that were impacted by the severity of illness in the hosts before and after HPV clearance.

Many studies have shown that increased cervicovaginal microbiota diversity is associated with advanced CIN lesions.^10^, ^14^, ^34^ In line with previous studies,^16^, ^17^ a reduced diversity in the cervicovaginal microbiota in CINs after treatment was found in our study (Figure 2). In contrast to previous reports that used a mixture of HPV‐positive and HPV‐negative samples and did not take into account the HPV type, we enrolled HPV16‐infected patients and showed that both antiviral and surgical treatments were effective for clearing HPV16 and restoring the cervical microbiota. Additionally, unlike prior studies, we classified the patients into four groups according to disease severity and found that microbial recovery had distinct bacterial peculiarities for different disease statuses.

Similar to the findings in our previous study^2^ on CST grouping, CSTs II and IV were not composed of the bacteria previously reported, while the remaining CSTs, including CSTs I, III, and V, were similar to those previously described.^10^, ^14^, ^35^ In terms of community structure, CSTs II and IV were more similar to state types IV and II, respectively.^2^ The highly consistent structure of CSTs in the two studies implies the reliability of the results. The dominant bacteria within CSTs II and IV, including *Burkholderia*, *G*. *vaginalis*, *Pseudomonas*, *E*. *coli*, *Atopobium*, *S. amnii*, and *Prevotella,* are related to the elicitation of bacterial vaginosis (BV) and have the potential to facilitate the occurrence of CIN.^28^, ^36^ Further analysis revealed that the microbiota of HPV16‐infected women with CIN or SCC seemed to be more associated with CST IV and less associated with CST II (Figure 3). The main member in CST II, *Burkholderia*, has been speculated to have a positive effect on the elicitation of inflammation or even CIN^36^; here, its existence in CIN I‐1, CIN II/III‐1, and SCC‐1 again verified its pathogenicity.

Interestingly, the CSTs tended to convert into CST III after clinical treatment (Figure 3A–D, Table S13). With the clearance of HPV16, the proportions of CSTs I and III increased from 36.85% (7/19), 19.04% (4/21), and 0% (0/4) to 78.95% (15/19), 66.67% (14/21), and 50.00% (2/4) in the CIN I, CIN II/III, and SCC groups, respectively, of which 63.16% (12/19), 47.62% (10/21), and 50.00% (2/4) belonged to CST III. Although CST III was dominated by *L. iners*, the definite role of this species is still unclear, as *L. iners* has been detected in either healthy women or women with cervical disease.^9^ In contrast, *L. crispatus*‐dominated CST I was more abundant in the HC group than in the CST III group (Figure 3E). *Lactobacillus crispatus,* as the most predominant bacterium in the human cervicovagina, is considered representative of a healthy cervicovaginal space.^37^, ^38^ Enrichment of this strain was found to correspond to the lowest level of inflammation.^39^ Therefore, we suggest that the recovery of *Lactobacillus* species may undergo a transitional period in which *L. iners* is first enriched, after which other species of *Lactobacillus,* such as *L. crispatus,* begin to accumulate, although the removal of inflammatory areas might also affect this process. In addition, 21.1% (4/19 for CIN I), 33.3% (7/21 for CIN II/III), and 50.0% (2/4 for SCC) of patients maintained their CSTs as CST II or IV, even though the abundance of *Lactobacillus* was increased (Figure S2, Table S13). As CSTs II and IV are mostly composed of bacteria associated with an unhealthy or dysbiotic cervicovaginal status, more time may be needed to change the CSTs in some patients.

Intriguingly, we found that different species of *Lactobacillus* can serve as potential microbiological markers for predicting health status disparities. *Lactobacillus* confers resistance to HPV infection and protects against colonization by overt pathogens.^35^ However, the specific role of *Lactobacillus* in microbial recovery during HPV infection remains unclear. In the present study, *L. gasseri*‐dominated CST V was observed only in the HC group (Figure 3), suggesting that *L. gasseri* is a signature bacterium that may exist only in women with normal cytology. Moreover, LEfSe analysis (Figure 4) at the species level revealed that *L. secaliphilus* and *L. iners* were potential biomarkers representing CIN I‐2 and CIN II/III‐2, respectively, in which the two bacteria might be predictors of recovery from low‐ and high‐grade cytological changes.

CIN I‐1‐specific *Prevotella* might favor the development of low‐grade cytological changes (Figure 4). *Prevotella*, originating from the mouth and vagina, contributes to an increased risk for delayed clearance of HPV infection, which negatively impacts women's health.^40^, ^41^ Recently, *Prevotella* was reported to be associated with the occurrence of HPV infection‐associated CIN II/III, while LEEP surgery can reduce its abundance.^42^ In addition to its presence in CIN I‐1, *Prevotella* was more abundant in CIN II/III‐1 samples than in CIN II/III‐2 samples, while an opposite trend was found between SCC‐1 and SCC‐2 samples. These results suggest that antiviral treatment or local excisional treatment can promote the efficiency of microbiota recovery in CIN I‐1 and CIN II/III‐1 patients, whereas the recovery ability is diminished once the neoplasia becomes invasive.

In addition to *Prevotella*, we found another two genera, *Atopobium* and *Aerococcus* that were unique to the SCC‐1 subgroup (Figure 4). *Atopobium* is an anaerobic bacterium that has been found in high titers in the upper genital tract of most women who have bacterial vaginosis.^43^ In addition, *Aerococcus* has been recognized as the most common human pathogen causing urinary tract infection and modulates the immune response by inducing the secretion of pro‐inflammatory cytokines associated with cervical lesions.^44^ It is reasonable to speculate that the existence of these two bacteria might be related to tumor formation after HPV16 infection.

One strength of our study is that it included patients with different disease stages before and after treatments, allowing us to comprehensively understand the linkage between cervicovaginal microbiota changes and clinical treatments. Another strength is that we restricted our study to only those patients singly infected with HPV16, which implies that the changes in the microbiota composition after clinical treatments are not confounded by other HPV subtypes. Despite these two strengths, a causal relationship between the metabolites and microbiota changes in the cervical microenvironment was not established. In addition, the sample size was relatively small for the SCC group due to the difficulty in obtaining tumor samples, which might have obscured the real differences between SCC‐1 and SCC‐2 samples (Figure 3D). Therefore, further studies combined with metabolome analysis using a larger number of patients will allow for the verification and extension of our findings.

## 5 CONCLUSION

In summary, we identified the differential cervicovaginal microbiota profiles and CSTs between HC women and HPV16‐infected women with CIN I, CIN II/III, or SCC. We also established the relationship between HPV16 clearance and cervicovaginal microbiota composition in each disease stage before and after clinical treatments. Nevertheless, further experimental biomolecular research is required to establish causation. Furthermore, our results suggest that both antiviral treatment and local excisional treatment are effective in clearing HPV16 infection and promoting the recovery of the cervicovaginal microbiota.

## CONFLICT OF INTEREST

The authors declare no conflict of interest.

## AUTHOR CONTRIBUTIONS

Y. Yang, Z. Zhang and H. Liao designed research; H. Liao, Z. Zhang, Y. Yang, and C. Li analyzed data; H. Liao, Z. Zhang and C. Li wrote the paper; Y. Yang and C. Li revised the paper.

## ETHICS STATEMENT

This study was approved by the Scientific and Ethical Committee of the Shanghai First Maternity and Infant Hospital affiliated with Tongji University (KS1895).

## Supporting information


**Appendix S1:** Supporting Information

## Data Availability

All raw sequences were deposited in the NCBI Sequence Read Archive under accession number PRJNA643773.
