# Supplementary material for: Changes in the cervicovaginal microbiota composition of HPV16‐infected patients after clinical treatment
Source: Cancer Med. 2022 May 15;11(24):5037–49. doi: 10.1002/cam4.4801 (PMC9761074; doi:10.1002/cam4.4801)
Supplement: Supplementary file 1 — Appendix S1: Supporting Information [file CAM4-11-5037-s001.zip › CAM4_4801_Supplementary Figures.docx]

Supplementary Material

# Supplementary Figures


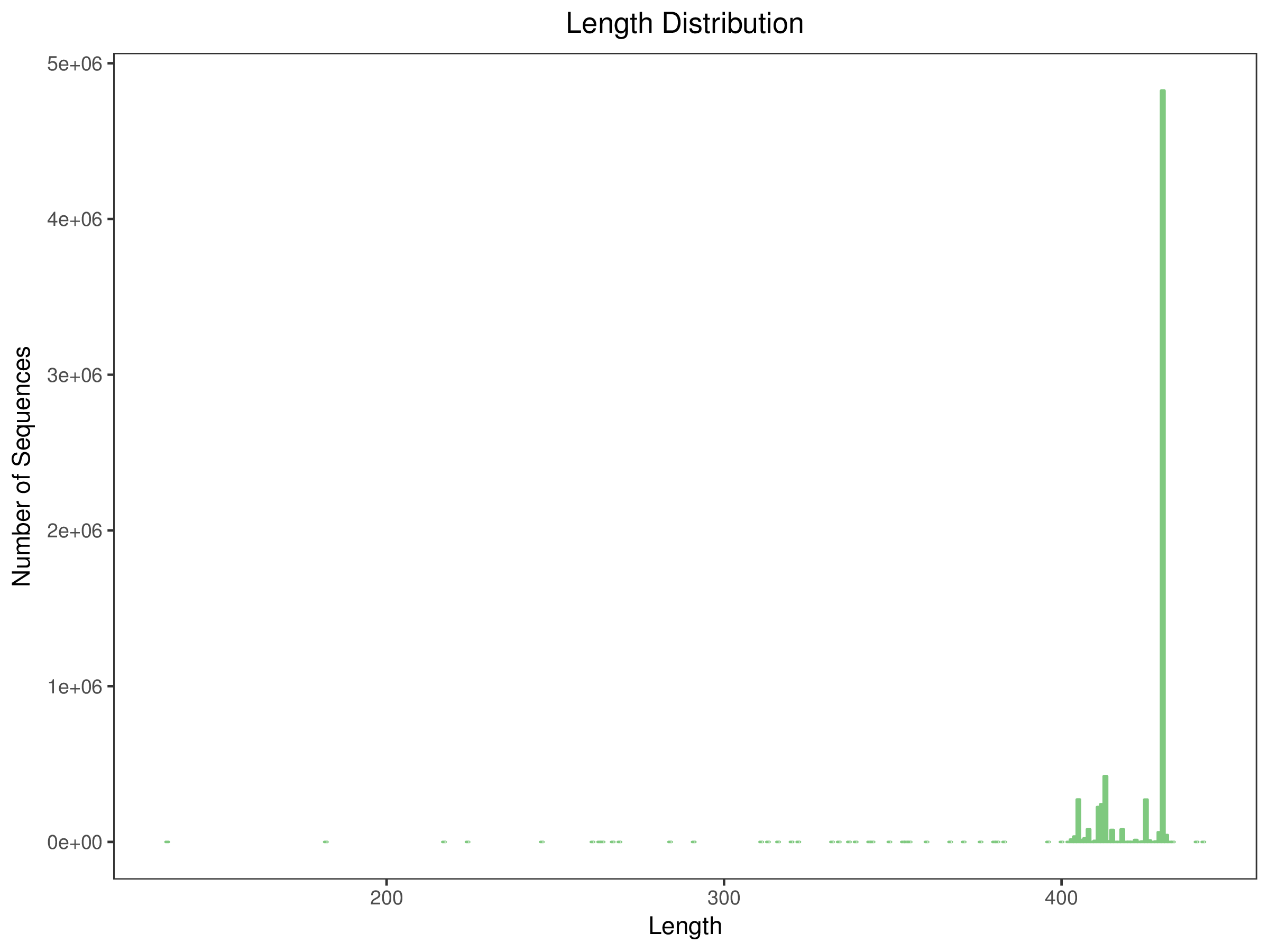


**Supplementary Figure 1.** Length distribution of sequences determined by Illlumina MiSeq pyrosequencing.


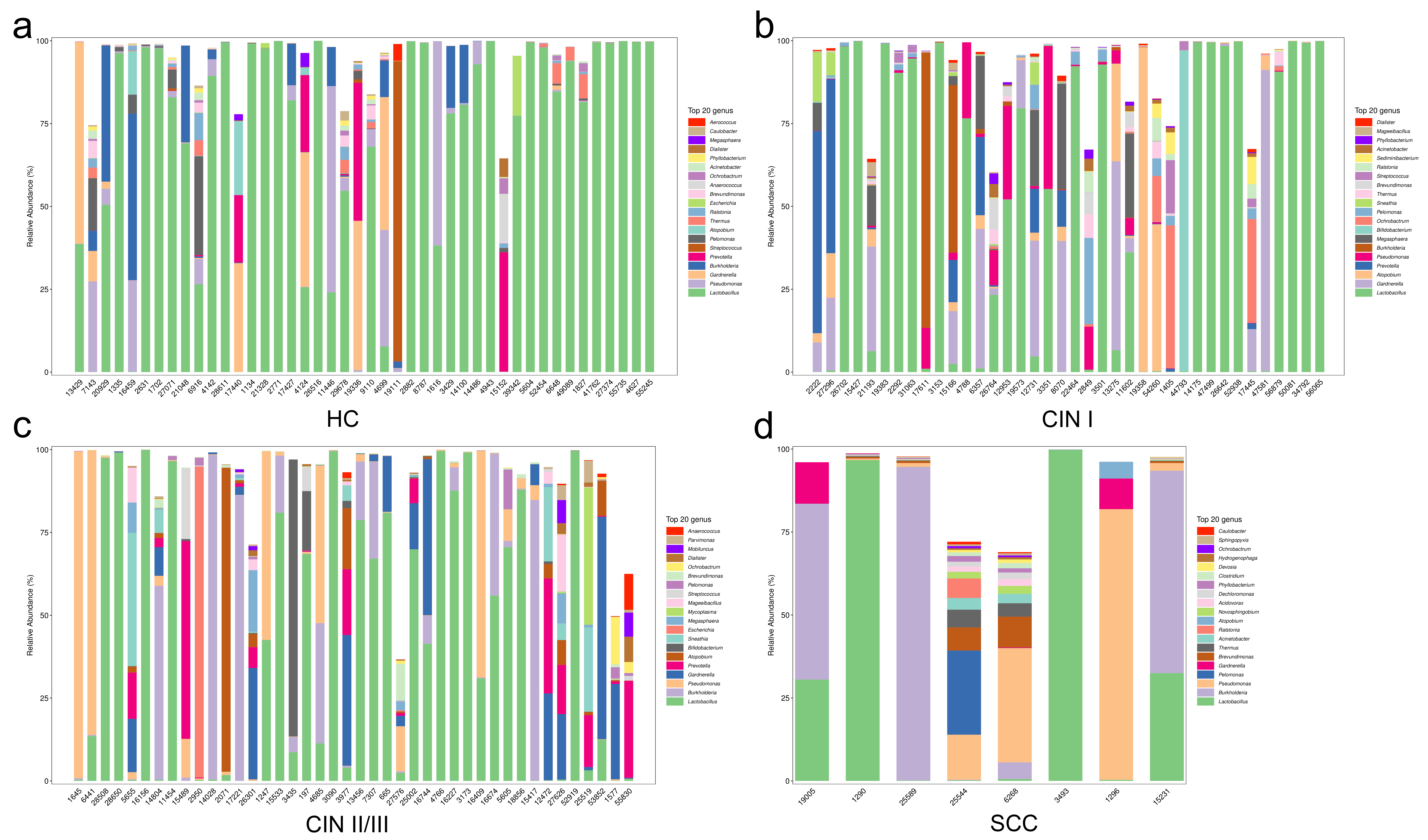


**Supplementary Figure 2.** Bacterial abundance of the samples in each group at the genus level. The top 20 genera were shown. (a) HC. (b) CIN I. (c) CIN II/III. (d) SCC. Paired samples were shown, for example in (a): 13429/7143, 20929/1335, 16459/2631, 1702/27071 represent microbiota distribution from four women before/after treatments.


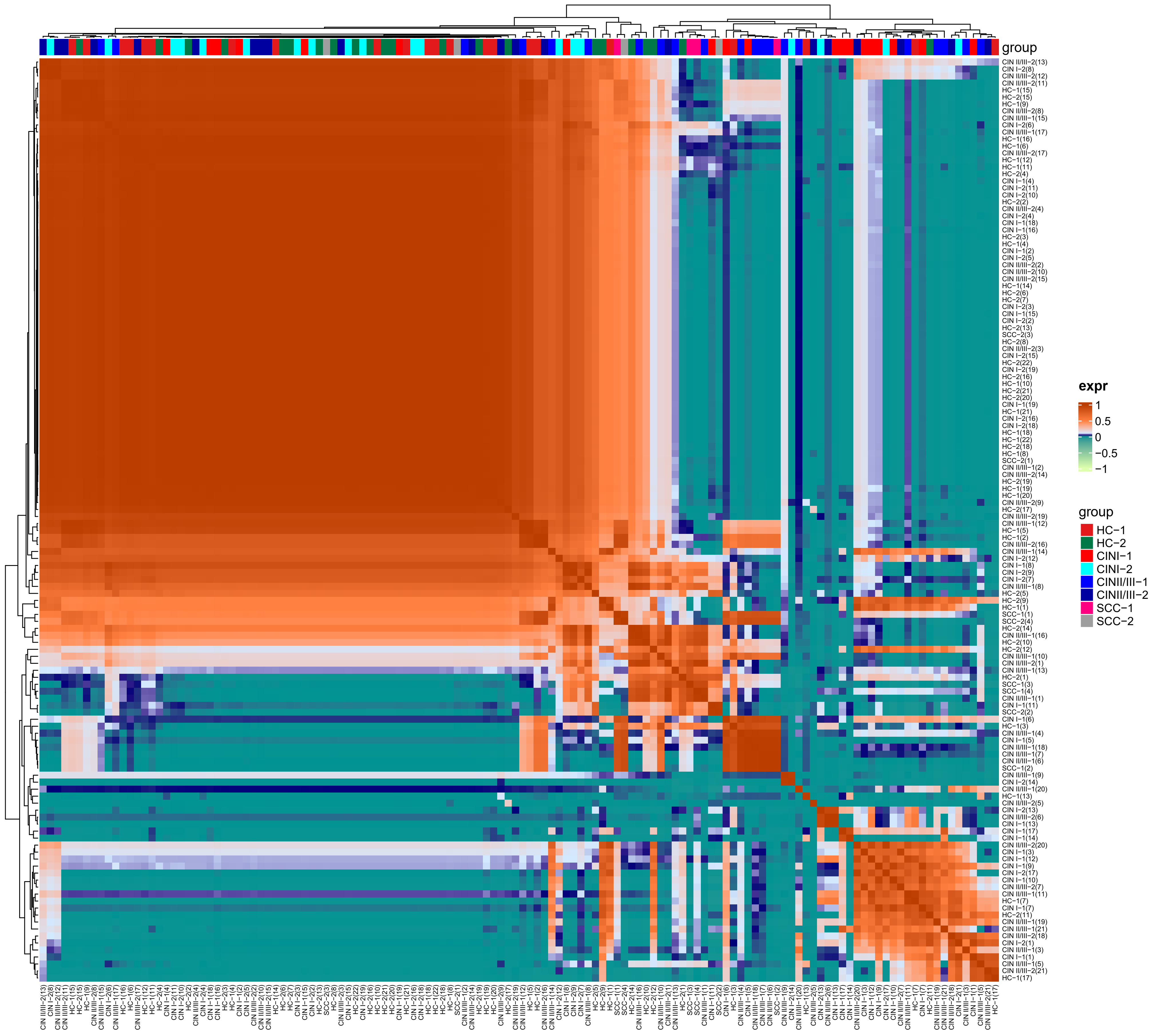


**Supplementary Figure 3.** Heatmap analysis of β diversity based on the genus profile to show pair-wise similarity of the subjects with hierarchical clustering. CIN I-1, CIN II/III-1 and SCC-1 indicate that the samples were collected before clinical treatment, CIN I-2, CIN II/III-2 and SCC-2 indicate that the samples were collected after clinical treatment. The numbers in brackets represent different patients. Statistical analysis was performed using RStudio v1.4.1717. Pearson correlation analysis was used to determine the correlation for pair-wise similarity of the subjects. A Mantel test was used to assess Pearson correlation. *p* < 0.05 was considered statistically significant.


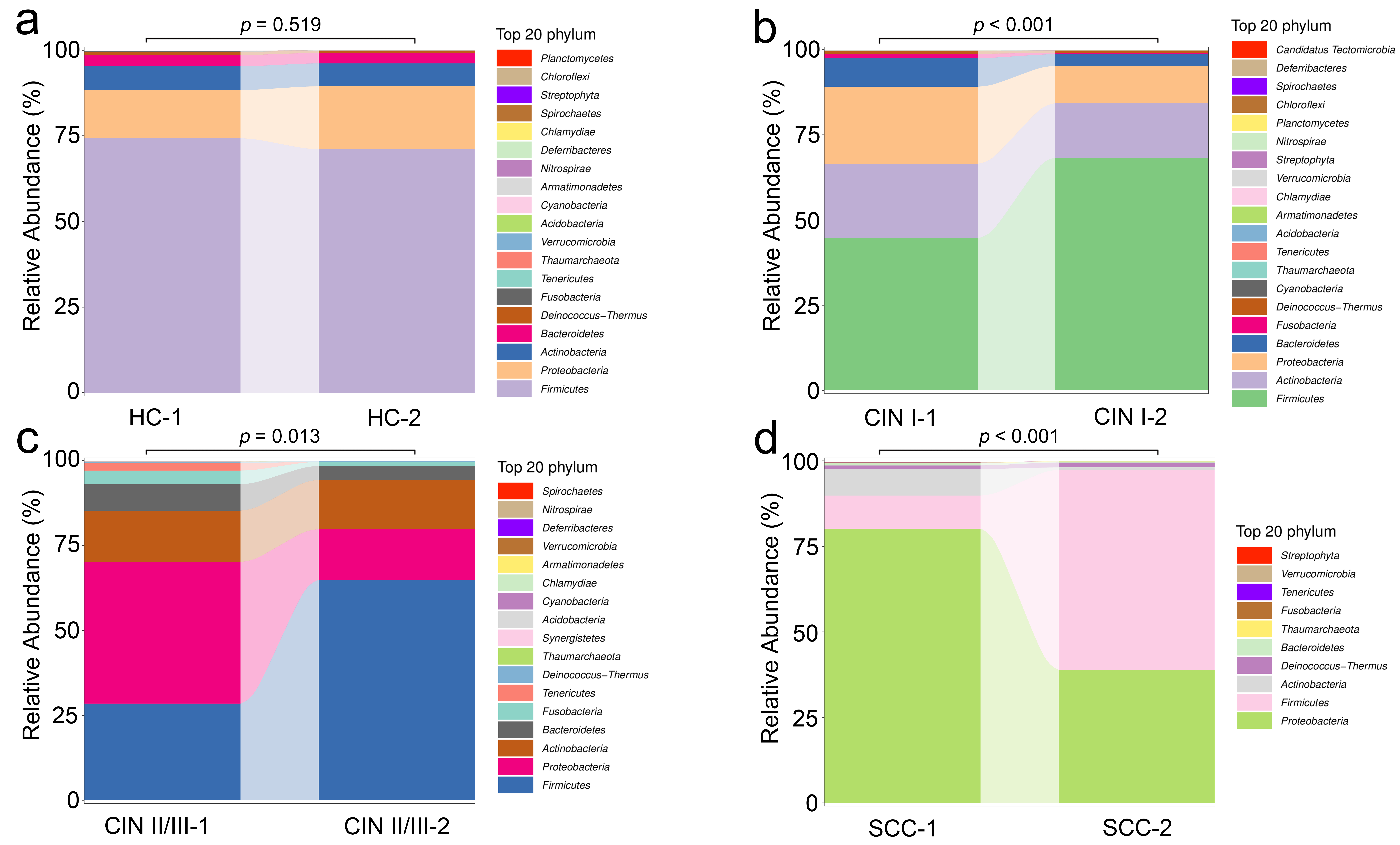


**Supplementary Figure 4.** The bacterial distribution and abundance in each group at the phyla level. The top 20 phyla were shown. (a) HC. (b) CIN I. (c) CIN II/III. (d) SCC. HC-1, CIN I-1, CIN II/III-1 and SCC-1 indicate the samples were collected before clinical treatment; HC-2, CIN I-2, CIN II/III-2 and SCC-2 indicate the samples were collected after clinical treatment. Statistical analysis was performed using SPSS 20.0, the chi-square test was used to evaluate differences in the phylum distribution among the groups. *p* < 0.05 was considered statistically significant.


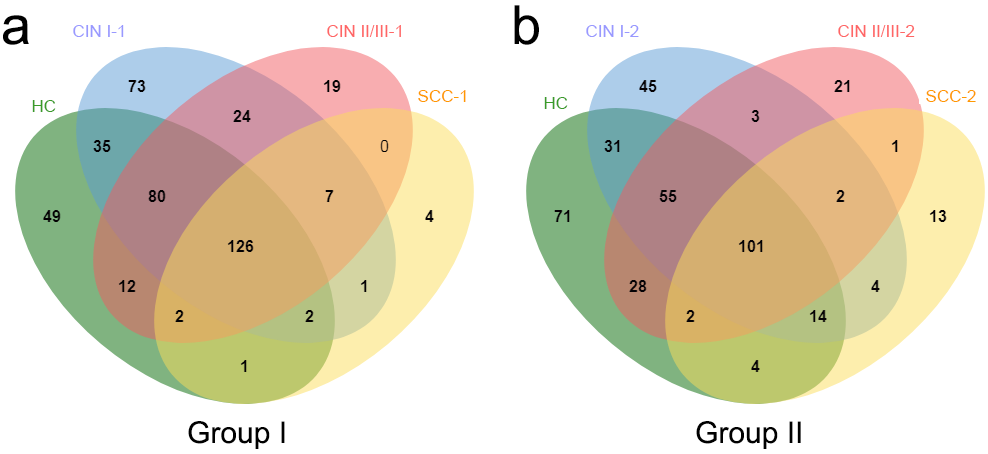


**Supplementary Figure 5.** Analysis of co-abundance bacteria enriched in each group. (a) A Venn diagram representing the shared and unique taxa within Group I at the genus level. (b) A Venn diagram representing the shared and unique taxa within Group II at the genus level.


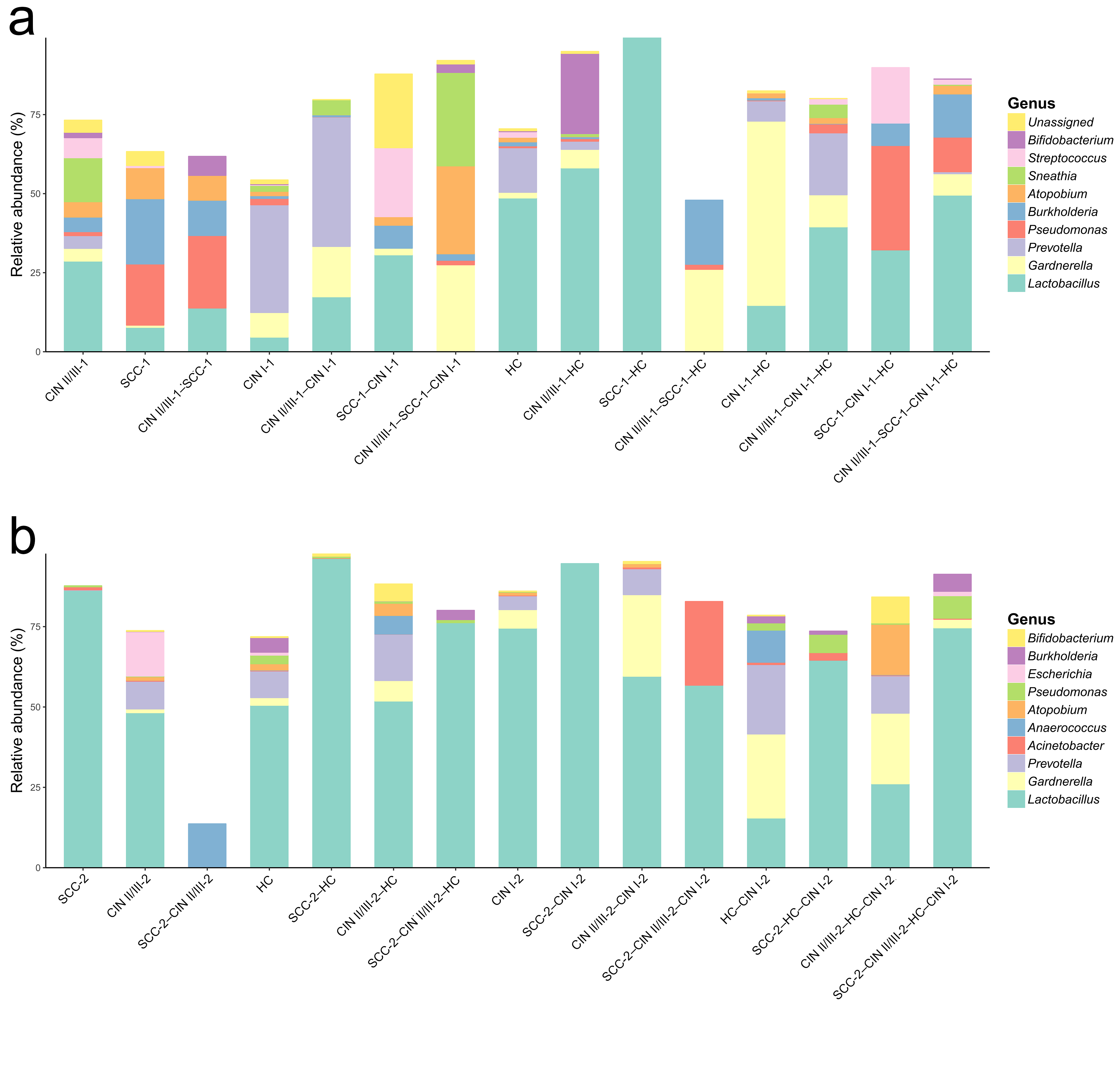


**Supplementary Figure 6.** Histogram of bacterial abundance at the genus level in different regions of Venn diagram shown in Fig. S5a and b, respectively.
